# Supplementary material for: The interactome of intact mitochondria by cross-linking mass spectrometry provides evidence for coexisting respiratory supercomplexes
Source: Mol Cell Proteomics. 2017 Dec 8;17(2):216–32. doi: 10.1074/mcp.RA117.000470 (PMC5795388; doi:10.1074/mcp.RA117.000470)
Supplement: Supplemental Data [file supp_RA117.000470_133922_0_supp_23978_fzffwf.zip › spectra_annotation/mito_DR_spectra_annotation/237-2-2-1-5-1.pdf]

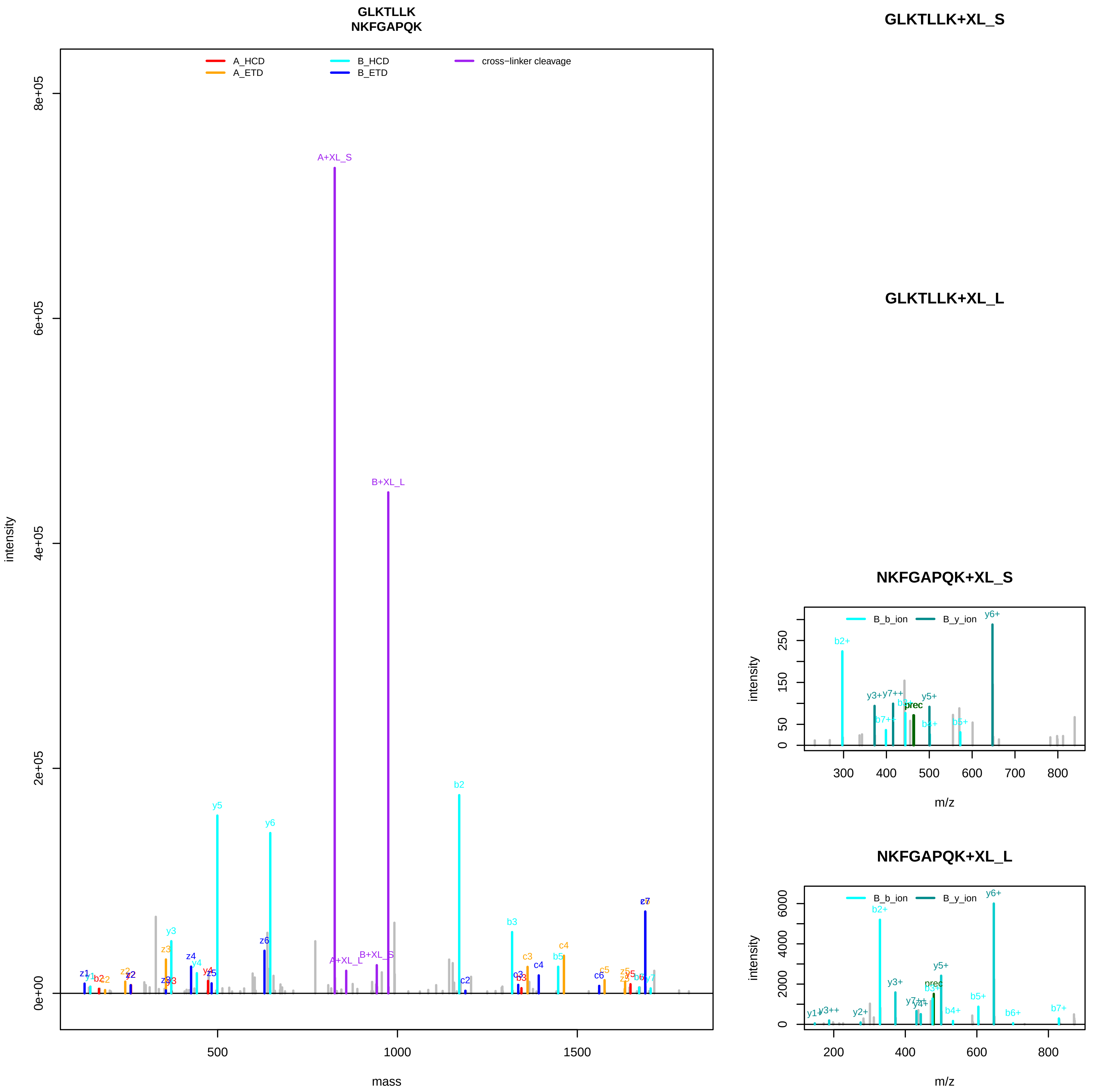

GLKTLLK
NKFGAPQK
A_HCD
A_ETD
B_HCD
B_ETD
cross−linker cleavage
intensity
mass
A+XL_S
B+XL_L
A+XL_L
B+XL_S
y5
y6
b2
b3
b5
c3
c4
c6
z6
z7
GLKTLLK+XL_S
GLKTLLK+XL_L
NKFGAPQK+XL_S
B_b_ion
B_y_ion
y6+
b2+
y3+
y7++
y5+
prec
m/z
NKFGAPQK+XL_L
b5+
b6+
b7+
y2+
